# Elucidating Syntrophic Butyrate-Degrading Populations in Anaerobic Digesters Using Stable-Isotope-Informed Genome-Resolved Metagenomics

**DOI:** 10.1128/mSystems.00159-19

**Published:** 2019-08-06

**Authors:** Ryan M. Ziels, Masaru K. Nobu, Diana Z. Sousa

**Affiliations:** a Department of Civil Engineering, University of British Columbia, Vancouver, British Columbia, Canada; b Department of Civil and Environmental Engineering, University of Washington, Seattle, Washington, USA; c Bioproduction Research Institute, National Institute of Advanced Industrial Science and Technology, Tsukuba, Japan; d Laboratory of Microbiology, Wageningen University & Research, Wageningen, Netherlands; University of Waterloo

**Keywords:** anaerobic catabolic pathways, anaerobic digestion, metagenomics, methanogenesis, stable-isotope probing, syntrophy

## Abstract

Linking the genomic content of uncultivated microbes to their metabolic functions remains a critical challenge in microbial ecology. Resolving this challenge has implications for improving our management of key microbial interactions in biotechnologies such as anaerobic digestion, which relies on slow-growing syntrophic and methanogenic communities to produce renewable methane from organic waste. In this study, we combined DNA stable-isotope probing (SIP) with genome-centric metagenomics to recover the genomes of populations enriched in ^13^C after growing on [^13^C]butyrate. Differential abundance analysis of recovered genomic bins across the SIP metagenomes identified two metagenome-assembled genomes (MAGs) that were significantly enriched in heavy [^13^C]DNA. Phylogenomic analysis assigned one MAG to the genus *Syntrophomonas* and the other MAG to the genus *Methanothrix.* Metabolic reconstruction of the annotated genomes showed that the *Syntrophomonas* genome encoded all the enzymes for beta-oxidizing butyrate, as well as several mechanisms for interspecies electron transfer via electron transfer flavoproteins, hydrogenases, and formate dehydrogenases. The *Syntrophomonas* genome shared low average nucleotide identity (<95%) with any cultured representative species, indicating that it is a novel species that plays a significant role in syntrophic butyrate degradation within anaerobic digesters. The *Methanothrix* genome contained the complete pathway for acetoclastic methanogenesis, indicating that it was enriched in ^13^C from syntrophic acetate transfer. This study demonstrates the potential of stable-isotope-informed genome-resolved metagenomics to identify *in situ* interspecies metabolic cooperation within syntrophic consortia important to anaerobic waste treatment as well as global carbon cycling.

**IMPORTANCE** Predicting the metabolic potential and ecophysiology of mixed microbial communities remains a major challenge, especially for slow-growing anaerobes that are difficult to isolate. Unraveling the *in situ* metabolic activities of uncultured species may enable a more descriptive framework to model substrate transformations by microbiomes, which has broad implications for advancing the fields of biotechnology, global biogeochemistry, and human health. Here, we investigated the *in situ* function of mixed microbiomes by combining stable-isotope probing with metagenomics to identify the genomes of active syntrophic populations converting butyrate, a C_4_ fatty acid, into methane within anaerobic digesters. This approach thus moves beyond the mere presence of metabolic genes to resolve “who is doing what” by obtaining confirmatory assimilation of the labeled substrate into the DNA signature. Our findings provide a framework to further link the genomic identities of uncultured microbes with their ecological function within microbiomes driving many important biotechnological and global processes.

## Abstract (precis)

Predicting the metabolic potential and ecophysiology of mixed microbial communities remains a major challenge, especially for slow-growing anaerobes that are difficult to isolate. Unraveling the *in situ* metabolic activities of uncultured species may enable a more descriptive framework to model substrate transformations by microbiomes, which has broad implications for advancing the fields of biotechnology, global biogeochemistry, and human health. Here, we investigated the *in situ* function of mixed microbiomes by combining stable-isotope probing with metagenomics to identify the genomes of active syntrophic populations converting butyrate, a C_4_ fatty acid, into methane within anaerobic digesters. This approach thus moves beyond the mere presence of metabolic genes to resolve “who is doing what” by obtaining confirmatory assimilation of the labeled substrate into the DNA signature. Our findings provide a framework to further link the genomic identities of uncultured microbes with their ecological function within microbiomes driving many important biotechnological and global processes.

## INTRODUCTION

Linking microbial genomic identity with ecological function is considered a “Holy Grail” in microbial ecology (1) and has broad implications for improving our ability to manage microbial communities in engineered biotechnologies. Anaerobic digestion is an example of a biotechnology that enables resource recovery from organic waste by generating methane gas as a renewable biofuel and thus plays a role in establishing a circular economy (2). The production of methane in anaerobic digestion is executed through a series of trophic interactions constituting a metabolic network of hydrolyzing and fermenting bacteria, syntrophic acetogens, and methanogenic archaea (3, 4). Metabolic reconstructions based on shotgun metagenomic sequencing data have highlighted potential partitioning of functional guilds within anaerobic digester microbiomes (4). Yet, our understanding of the ecophysiology of the microorganisms present in anaerobic digesters is limited by the high community complexity and lack of cultured representatives (4). Elucidating the nature of interspecies interactions between different trophic groups in the anaerobic digester metabolic network may help to better understand and optimize the conversion of organic wastes into renewable methane.

The terminal steps in the anaerobic metabolic network, syntrophy and methanogenesis, are responsible for a considerable portion of carbon flux in methanogenic bioreactors, as fatty acids are often produced during fermentation of mixed organic substrates (5). The accumulation of fatty acids in anaerobic digesters is often responsible for a reduction in pH and process instability (3). In particular, syntrophic degradation of the 4-carbon fatty acid butyrate can be a bottleneck for anaerobic carbon conversion, as this metabolism occurs at the thermodynamic extreme. Butyrate degradation to acetate and hydrogen is thermodynamically unfavorable under standard conditions (Δ*G*° = 53 kJ/mol) and yields only −21 kJ/mol under environmental conditions typical of anaerobic bioreactors (pH 7, 1 mM butyrate and acetate, 1 Pa H_2_) (see equation S1 in Table S2 in the supplemental material). Thus, cooperation between syntrophic bacteria and acetate- and hydrogen-scavenging methanogenic partners is necessary to maintain thermodynamic favorability. Cultured representative species carrying out syntrophic fatty acid oxidation are potentially underrepresented due to their slow growth and difficulty of isolation in the lab (6). So far, only two mesophilic (*Syntrophomonas* and *Syntrophus*) and two thermophilic (*Syntrophothermus* and *Thermosyntropha*) genera (12 bacterial species in total) have been shown to oxidize butyrate in syntrophic cooperation with methanogenic archaea, and they all belong to the families *Syntrophomonadaceae* and *Syntrophaceae* (6). Despite their major roles in processing carbon within anaerobic bioreactors, many syntrophic fatty acid-oxidizing bacteria have evaded detection with quantitative hybridization-based techniques (7), which is likely due to their low biomass yields (8) or our incomplete knowledge of active syntrophic populations within anaerobic digesters (9). Broad metagenomic surveys of anaerobic digester communities have similarly observed poor resolution of syntrophic populations, owing to their low abundance (4, 10). Thus, highly sensitive culture-independent approaches are needed to expand our understanding of the ecophysiology of syntrophic populations to better control and predict metabolic fluxes in anaerobic environments.

Recently, we demonstrated the potential of combining DNA–stable-isotope probing (DNA-SIP) with genome-resolved metagenomics to identify syntrophic populations degrading the long-chain fatty acid oleate (C_18:1_) within anaerobic digesters (11). Stable-isotope-informed metagenomic sequencing can enrich metagenomic libraries with genomic sequences of actively growing microbes that incorporate ^13^C into their biomass from an added labeled substrate (12) and thus allows for a “zoomed-in” genomic view of low-abundance populations, such as syntrophs. We also demonstrated that this approach was amenable for recovering high-quality microbial genomes using a differential coverage-based binning approach, as genomes from active microbes have low abundance in heavy DNA from ^12^C controls but are enriched in heavy DNA from ^13^C-amended treatments (11). Here, we applied stable-isotope-informed metagenomics to resolve the genomic makeup of active syntrophic butyrate-degrading populations within anaerobic digesters treating manure and sodium oleate (13). These same anaerobic digesters were previously used for DNA-SIP with oleate (11) at a similar time point, thus allowing for genomic comparisons using a multisubstrate SIP data set. This approach identified potential metabolic flexibility in a syntrophic bacterium implicated in the degradation of multiple fatty acids within the study anaerobic digesters, and elucidated an *in situ* syntrophic partnership between the acetogenic bacterium and an acetoclastic methanogen via interspecies metabolite transfer during butyrate degradation.

## RESULTS AND DISCUSSION

### DNA-SIP of methanogenic microcosms with [^13^C]butyrate.

Two laboratory-scale anaerobic digesters fed dairy manure were either pulse fed every 48 h or fed semicontinuously with sodium oleate (C_18:1_) for over 230 days (13). Quantitative PCR and 16S rRNA gene amplicon sequencing indicated that *Syntrophomonas* became enriched within the reactors from oleate feeding (13). DNA-SIP-informed metagenomics confirmed that a majority of oleate-degrading bacteria in the two digesters belonged to *Syntrophomonas* (11). Here, we investigated whether any of the populations implicated in oleate degradation were also involved in the degradation of the short-chain fatty acid butyrate (C_4_). Digestate from the pulse-fed and continuously fed anaerobic digester were incubated in duplicate microcosms, which were spiked with either [^12^C]- or [^13^C]butyrate (40 mM) for approximately 50 h. The added butyrate was converted into methane at a >80% conversion efficiency based on chemical oxygen demand (COD) recovery (see Fig. S1 in the supplemental material). After the 50-h incubation, the contents of the microcosms were sacrificed for DNA extraction, density gradient centrifugation, and fractionation.

10.1128/mSystems.00159-19.1 FIG S1 Cumulative methane production (minus blank controls) for the microcosms fed with ^12^C- and ^13^C-labeled butyrate over approximately 50 h. The black dashed line shows the theoretical methane potential of the added butyrate (25.3 ml CH_4_; based on 1.82 g COD/g butyrate, 40 mM concentration, 10 ml sample, and 35°C temperature). Error bars represent the standard deviations of results from the biological replicates. Download FIG S1, PDF file, 0.01 MB. Copyright © 2019 Ziels et al. 2019 Ziels et al. This content is distributed under the terms of the Creative Commons Attribution 4.0 International license.

The abundance of 16S rRNA genes of the known butyrate-degrading genus *Syntrophomonas* was quantified across density gradient fractions using quantitative PCR (qPCR) to identify DNA fractions that were enriched in ^13^C. Density fractions with a buoyant density from 1.70 to 1.705 had 2.0 to 2.2 times more *Syntrophomonas* 16S rRNA genes (normalized to the maximum concentration) than the ^12^C controls (Fig. S2). Those DNA fractions were selected from each SIP microcosm for metagenomic sequencing, as well as for 16S rRNA gene amplicon sequencing.

10.1128/mSystems.00159-19.2 FIG S2 Ratios of *Syntrophomonas* 16S rRNA genes measured by qPCR in each density gradient fraction to the maximum observed across all density fractions. The points indicate the average values of the biological duplicates for ^13^C-incubated microcosms and ^12^C-labeled controls for both anaerobic digesters. The widths of error bars indicate the range of the biological duplicates. The filled points indicate density gradient fractions that were pooled for subsequent 16S rRNA gene amplicon sequencing and metagenomic sequencing. Download FIG S2, PDF file, 0.03 MB. Copyright © 2019 Ziels et al. 2019 Ziels et al. This content is distributed under the terms of the Creative Commons Attribution 4.0 International license.

**FIG 1:**
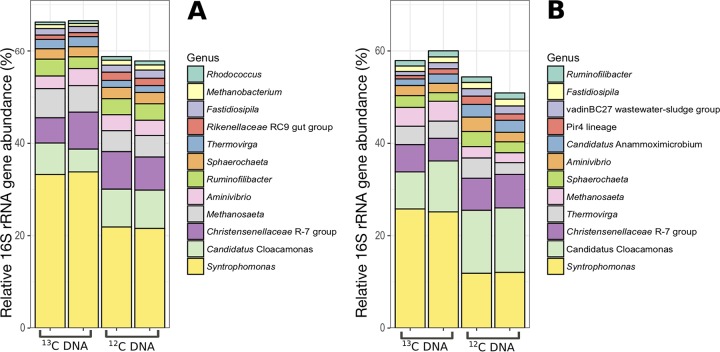
Relative 16S rRNA gene amplicon sequence abundances of the top 12 most abundant prokaryotic genera in heavy DNA from [^13^C]butyrate-amended microcosms and the [^12^C]butyrate-amended controls for the pulse-fed codigester (A) and the continuously fed codigester (B). Values for each biological duplicate are shown for each condition (^12^C or ^13^C) for both anaerobic digesters.

The microbial communities in the heavy density gradient fractions were assessed through paired-end 16S rRNA gene amplicon sequencing for all ^12^C- and ^13^C-incubated duplicate microcosms (Fig. 1). Differential abundance analysis of operational taxonomic unit (OTU) read counts with DESeq2 (14) showed that approximately 50% (7 of 15) of the significantly enriched (*P < *0.05) OTUs in heavy [^13^C]DNA samples relative to heavy [^12^C]DNA were taxonomically classified as *Syntrophomonas* for the pulse-fed digester (Fig. S3). For the continuously fed digester, approximately 40% of the ^13^C-enriched OTUs (7 of 17) were assigned to *Syntrophomonas* (Fig. S3). Additionally, two ^13^C-enriched OTUs in both digesters were assigned to *Methanothrix* (formerly *Methanosaeta*), which likely scavenges the [^13^C]acetate generated by *Syntrophomonas* during [^13^C]butyrate degradation. While one previous study observed that *Syntrophaceae* was enriched predominantly in anaerobic digester granular sludge incubated with [^13^C]butyrate (9), various other studies also detected *Syntrophomonadaceae* populations (i) as active syntrophic butyrate degraders in anaerobic digester sludge using [^14^C]butyrate and microautoradiography–fluorescent *in situ* hybridization (MAR-FISH) (15), (ii) in anaerobic digester sludge by means of SIP using [^13^C]oleate (11), and (iii) in rice paddy soil with SIP using [^13^C]butyrate (16). In the last two studies, acetate-scavenging partners (*Methanothrix* and *Methanosarcinaceae*) were also enriched. Indeed, syntrophic interaction with acetoclastic methanogens is beneficial, as acetate accumulation can thermodynamically hinder butyrate oxidation (e.g., the Δ*G* exceeds the theoretical threshold for catabolism (–10 kJ/mol) when acetate accumulates beyond 10 mM (pH 7, 1 mM butyrate, and 1 Pa H_2_) (assumptions appear in Table S2). Notably, H_2_- and formate-consuming methanogens necessary for syntrophy were not detected during degradation of [^13^C]butyrate, likely because these archaea utilize CO_2_ as a carbon source and no [^13^C]CO_2_ is produced during butyrate oxidation.

10.1128/mSystems.00159-19.3 FIG S3 The log_2_-fold change in abundance of 16S rRNA amplicon OTUs in the pulse-fed codigester (A) and the continuously fed codigester (B) that were identified as significantly enriched in ^13^C samples versus ^12^C samples using DESeq2 (14), along with their genus- and phylum-level taxonomic assignments. Each point represents a ^13^C-enriched OTU. The significantly enriched OTUs were detected based on their abundance in duplicate ^13^C samples relative to duplicate ^12^C samples. Download FIG S3, PDF file, 0.03 MB. Copyright © 2019 Ziels et al. 2019 Ziels et al. This content is distributed under the terms of the Creative Commons Attribution 4.0 International license.

Our results also found ^13^C-enriched OTUs from lineages not known to degrade butyrate under methanogenic conditions: *Treponema*, *Luteimonas*, *Thauera*, *Christensenellaceae* (*Firmicutes*), and *Anaerolineaceae* (*Chloroflexi*) (Fig. S3). Other studies using [^13^C]butyrate also detected enrichment of populations likely unable to degrade butyrate, including *Tepidanaerobacter* and *Clostridium*, in a thermophilic anaerobic digester operated at 55°C (9) and *Chloroflexi* and *Planctomycetes* in rice paddy soil (16). Members of *Tepidanaerobacter* and *Clostridium* are known to syntrophically oxidize acetate under thermophilic conditions (17) and may have thus been enriched in [^13^C]RNA from [^13^C]acetate produced during the beta-oxidation of labeled butyrate in the study by Hatamoto et al. (9). Similarly, the *Chloroflexi* and *Planctomycetes* populations were hypothesized to have become enriched due to cross-feeding of intermediate metabolites, like acetate, in rice paddy soil (16). Thus, the “peripheral” populations detected in our study may grow on cellular-decay products, as genome-resolved metagenomics recently indicated that some uncultured *Anaerolineaceae* species are likely fermenters in anaerobic digesters (18). These results thus suggest that carbon cross-feeding may occur between multiple microbial trophic groups during the syntrophic degradation of butyrate in anaerobic digesters.

10.1128/mSystems.00159-19.4 FIG S4 Heatmaps of average nucleotide identity (ANI) between genomes from *Syntrophomonadaceae* (A) and *Methanosarcinales* (B). Genomes were clustered based on the ANI values using Ward’s minimum-variance method. The genome names shown in bold were identified in this study. Other genomes were obtained via the NCBI nr database (downloaded April 2018). Download FIG S4, PDF file, 0.1 MB. Copyright © 2019 Ziels et al. 2019 Ziels et al. This content is distributed under the terms of the Creative Commons Attribution 4.0 International license.

### Identifying active metagenome-assembled genomes (MAGs) in SIP metagenomes.

Metagenomic sequencing of heavy DNA from duplicate [^13^C]- and [^12^C]butyrate-amended microcosms yielded an average of 30 million paired reads per sample for both digesters (*n *=* *8) (Table S1). The filtered reads from heavy [^13^C]DNA were coassembled, yielding a total assembly length of 516 Mb of contigs larger than 1 kb, with an average (*N*_50_) contig length of 5 kb. The fraction of filtered short reads that mapped to the coassembly were 66% ± 3% (standard deviation) and 69% ± 1% for the ^12^C- and ^13^C-labeled metagenomes, respectively (*n *=* *4 each) (Table S1). The coassembly generated from ^13^C reads thus captured much of the genomic information present in the heavy-DNA fractions.

10.1128/mSystems.00159-19.5 TABLE S1 Summary of SIP metagenomes utilized for the coassembly, including the number of raw and filtered reads and the fraction of reads that mapped to coassembly. Download Table S1, DOCX file, 0.01 MB. Copyright © 2019 Ziels et al. 2019 Ziels et al. This content is distributed under the terms of the Creative Commons Attribution 4.0 International license.

The assembled metagenomic contigs were organized into 160 genomic bins at various levels of completion and redundancy (Data Set S1). Differential abundance analysis of the mapped read counts for the bins across the ^13^C- and ^12^C-labeled metagenomes with DESeq2 (14) identified two genomic bins that were significantly (*P < *0.05) enriched in [^13^C]DNA (Table 1). These genomic bins were enriched in [^13^C]DNA in both the pulse-fed and continuously fed bioreactors. Based on suggested completion and redundancy metrics for MAGs (19), one genomic bin is classified as a high-quality MAG (completion, >90%; redundancy, <10%), while the other is a medium-quality MAG (completion, >50%; redundancy, <10%). Taxonomic classification with CheckM (20) assigned one of the MAGs to the genus *Syntrophomonas* and the other to *Methanothrix* (Table 1).

**TABLE 1:** Genomic feature summary of the two metagenome-assembled genomes that were significantly enriched in [^13^C]DNA after the degradation of [^13^C]butyrate

Name	Bin ID	Taxonomy^*a*^	Size (Mb)	GC (%)	Completion (%)^*b*^	Redundancy (%)^*b*^
*Syntrophomonas* BUT1	Bin 26_1	*Syntrophomonas*	2.87	51.2	96.4	1.4
*Methanothrix* BUT2	Bin 26_2	*Methanothrix*	1.44	53.6	74.7	3.1

a Based on phylogenetic placement of single marker genes with CheckM (20).

b Measured with anvi’o (71).

10.1128/mSystems.00159-19.7 DATA SET S1 Summary of all genomic bins identified in the DNA-SIP metagenomes, including genome size, completeness, redundancy, coverage, and taxonomy. Download Data Set S1, XLSX file, 0.07 MB. Copyright © 2019 Ziels et al. 2019 Ziels et al. This content is distributed under the terms of the Creative Commons Attribution 4.0 International license.

**FIG 2:**
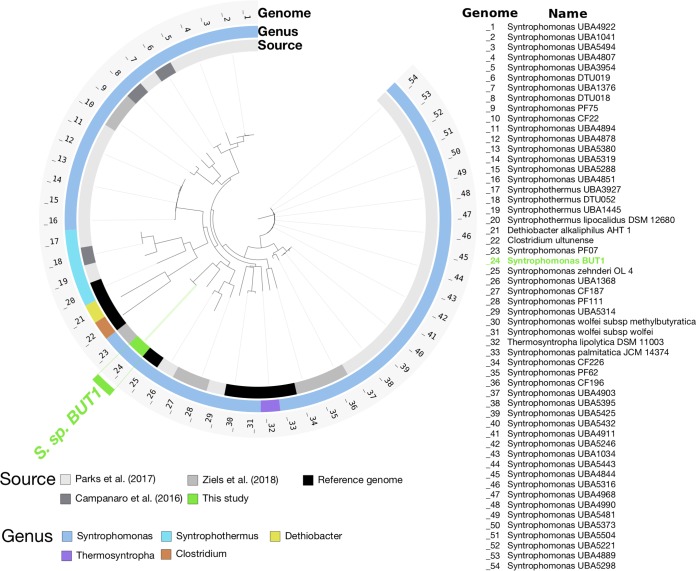
Phylogenomic tree showing the relationship of ^13^C-enriched *Syntrophomonas* BUT1 to other genomes available from the *Syntrophomonadaceae* family in the NCBI nr database (downloaded April 2018). The tree is based on a concatenated alignment of 139 bacterial single-copy marker genes (77) obtained using anvi’o (74). Open reading frames were predicted with Prodigal v.2.6.3 (70) and queried against sequences in a database of bacterial single-copy marker genes using HMMER v.2.3.2 (81). The tree was calculated using FastTree (82). The Clostridium ultunense genome was used as the outgroup.

The phylogenomic placement of the ^13^C-enriched *Syntrophomonas* BUT1 MAG was consistent with its taxonomic assignment, as it was located in the *Syntrophomonas* genome cluster within the family *Syntrophomonadaceae* (Fig. 2). The closest relative to *Syntrophomonas* BUT1 based on single-copy marker genes was *Syntrophomonas* PF07, which is a genomic bin enriched in ^13^C from DNA-SIP with labeled oleate (^13^C_18:1_) using sludge from the same pulse-fed digester used in this study (11). A high average nucleotide identity (ANI) of 99% was observed between the *Syntrophomonas* BUT1 and *Syntrophomonas* PF07 genomes (Fig. S4), suggesting that these two organisms likely originated from the same sequence-discrete population (21). The next-closest relative of *Syntrophomonas* BUT1 based on the phylogenomic analysis was Syntrophomonas zehnderi OL-4 (Fig. 2), which was isolated from an oleate-fed anaerobic granular sludge bioreactor (22). However, the ANI between *Syntrophomonas* BUT1 and Syntrophomonas zehnderi OL-4 was below 95% (Fig. S4), suggesting that these two organisms are different species (23). Thus, the active butyrate-degrading bacterial MAG identified in this study is distinct from any species obtained by isolation at this time. The detection of the sequence-discrete population of *Syntrophomonas* BUT1 within heavy [^13^C]DNA from both experiments with universally labeled butyrate and oleate indicates that this syntrophic population may be metabolically flexible; that is, it may grow on fatty acids of various lengths and degrees of saturation. An alternative explanation may be that *Syntrophomonas* BUT1 was detected in the SIP experiment with universally labeled [^13^C]oleate due to its degradation of shorter fatty acids, such as butyrate, excreted during oleate degradation by other community members. These findings have implications for current frameworks for mathematical modeling of anaerobic digesters, which typically assume that long-chain fatty acid (LCFA)-degrading and butyrate-degrading populations are distinct and do not cross-feed (24). Thus, the incorporation of genomic and functional characterization, as obtained through DNA-SIP genome-resolved metagenomics, may help to improve our ability to accurately model anaerobic digestion processes by accounting for metabolic flexibility or cross-feeding within key functional guilds.

**FIG 3:**
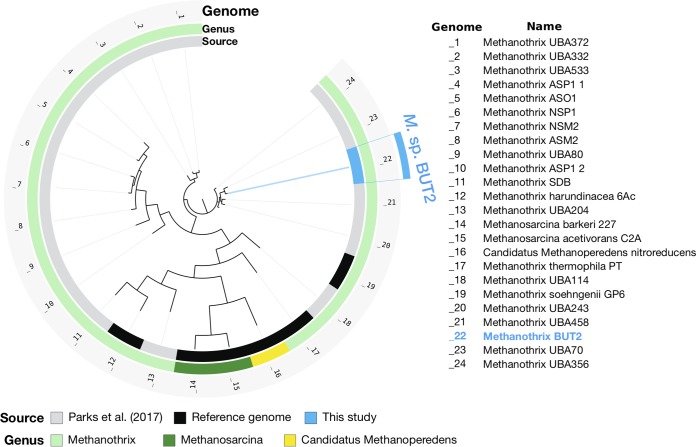
Phylogenomic tree showing the relationship of the ^13^C-enriched *Methanothrix* BUT2 to other genomes within the order *Methanosarcinales* in the NCBI nr database (downloaded April 2018). The tree is based on a concatenated alignment of 162 archaeal single-copy marker genes (78) obtained using anvi’o (74). Open reading frames were predicted with Prodigal v.2.6.3 (70) and queried against sequences in a database of archaeal single-copy marker genes using HMMER v.2.3.2 (81). The tree was calculated using FastTree (82). The “*Candidatus* Methanoperedens nitroreducens” genome was used as the outgroup.

A phylogenomic analysis of the ^13^C-enriched *Methanothrix* BUT2 MAG based on archaeal single-copy marker genes placed the MAG within the genus *Methanothrix*, consistent with its taxonomic assignment with CheckM (Fig. 3). *Methanothrix* BUT2 was closely clustered with the genome of Methanothrix soehngenii GP6, along with four MAGs reported in the study of Parks et al. (25). Congruently with the phylogenomic analysis, *Methanothrix* BUT2 shared an ANI of over 98% with Methanothrix soehngenii GP6 and the same with four MAGs from the work of Parks et al. (25) (*Methanothrix* UBA243, *Methanothrix* UBA458, *Methanothrix* UBA70, *Methanothrix* UBA356), indicating that these genomes likely form a sequence-discrete population (Fig. S4). A second, closely related population, including three MAGs from the work of Parks et al. (25) (*Methanothrix* UBA372, *Methanothrix* UBA332, *Methanothrix* UBA533) shared an ANI of 96% with the *Methanothrix* BUT2 population (Fig. S4).

DNA-SIP using [^13^C]oleate with the same anaerobic digester biomass as in this study did not identify any ^13^C-enriched methanogenic archaea in the genome-resolved metagenomic analysis (11). One possible explanation for the higher relative enrichment of methanogens on [^13^C]butyrate than on [^13^C]oleate may be the higher fraction of overall free energy partitioned toward methanogens during anaerobic butyrate degradation than during oleate degradation. For the overall conversion of 1 mol of butyrate to CO_2_ and CH_4_ under environmental conditions in anaerobic digesters, the thermodynamic yields would be −21.1, −9.4, and −58.9 kJ for the acetogenic bacteria, hydrogenotrophic methanogens, and acetoclastic methanogens, respectively (Table S2). For a similar conversion of 1 mol of oleate, the thermodynamic yields would be −219.9, −70.6, and −264.9 kJ, respectively (Table S2). Thus, the acetogen would gain a lower percentage of the overall free energy yield from the conversion of butyrate (24%) than from that of oleate (40%). As cell yield can depend on free energy (26), the lower yield of the butyrate degradation likely leaves a higher fraction of acetate for assimilation by an acetoclastic methanogen. The relative growth yields may also be particularly relevant due to the compositional nature of genome abundance data from the DNA-SIP metagenomes. As the stable-isotope-informed analysis utilized in this study depended on incorporation of the added ^13^C into biomass, it was not expected that autotrophic (i.e., hydrogenotrophic) methanogens would be highly enriched in the heavy [^13^C]DNA because no CO_2_ is produced during butyrate beta-oxidation and microcosms were preflushed with N_2_-[^12^C]CO_2_ (Table S2). Comparing the enriched communities from DNA SIP with different fatty acids, along with bicarbonate, may highlight differences in energy partitioning between syntrophic bacteria and different archaeal partners.

10.1128/mSystems.00159-19.6 TABLE S2 Gibbs free energy for some of the acetogenic and methanogenic reactions likely involved in the syntrophic conversion of butyrate and oleate. Download Table S2, DOCX file, 0.01 MB. Copyright © 2019 Ziels et al. 2019 Ziels et al. This content is distributed under the terms of the Creative Commons Attribution 4.0 International license.

### Metabolic potential of ^13^C-enriched MAGs.

Functional annotation and metabolic reconstruction of the ^13^C-enriched MAGs revealed their capacity to metabolize the [^13^C]butyrate into methane through syntrophic cooperation.

**FIG 4:**
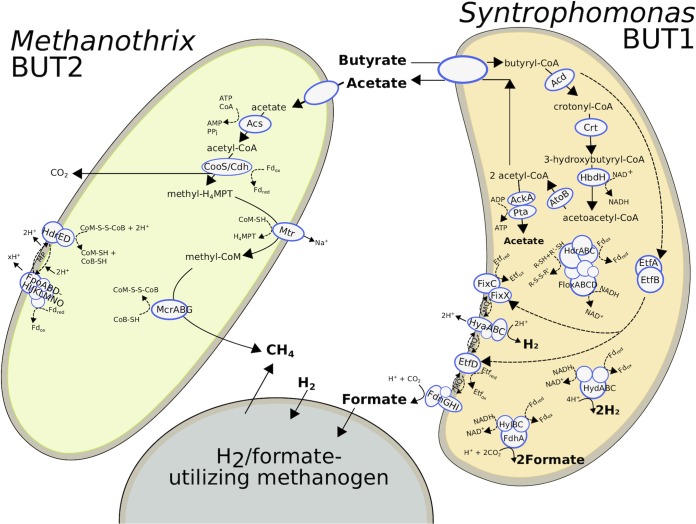
Cell diagram showing proposed metabolic pathways for anaerobic butyrate degradation in syntrophic cooperation between *Syntrophomonas* BUT1 and *Methanothrix* BUT2. The H_2_/formate-utilizing methanogenic partner is shown for conceptual purposes but was not identified with [^13^C]DNA-SIP in this study due to its autotrophic growth in the microcosms. Dotted lines indicate the direction of electron flow. Details of predicted proteins are given in Data Sets S2 and S3. Enzyme abbreviations are as follows: Fd, ferredoxin; (*Syntrophomonas* BUT1) Acd, acyl-CoA dehydrogenase; Crt, enoyl-CoA hydratase; HbdH, 3-hydroxybutyryl-CoA dehydrogenase; AtoB, acetyl-CoA acetyltransferase; AckA, acetate kinase; Pta, phosphate acetyltransferase; EtfA, electron transfer flavoprotein A; EtfB, electron transfer flavoprotein B; EtfD, EtfAB:quinone oxidoreductase; HydABC, bifurcating [Fe-Fe] hydrogenase; HyaABC, [NiFe] hydrogenase; FdhA-HylBC, formate dehydrogenase (electron bifurcating); FdnGHI, formate dehydrogenase (membrane bound, quinone reducing); FixC, electron transfer flavoprotein dehydrogenase; FixX, FixABC-associated ferredoxin; (*Methanothrix* BUT2) Acs, acetyl-coenzyme A synthetase; CooS, carbon monoxide dehydrogenase; CdhA, acetyl-CoA decarbonylase/synthase complex; Mtr, methyltetrahydromethanopterin:CoM methyltransferase; McrABG, methyl-coenzyme M reductase; HdrED, coenzyme B-coenzyme M heterodisulfide reductase; FpoABDHIJKLMNO, F_420_H_2_ dehydrogenase.

A complete pathway for butyrate β-oxidation was annotated for *Syntrophomonas* BUT1, indicating that this MAG was capable of metabolizing the added [^13^C]butyrate (Fig. 4). Notably, several homologues were detected for genes in the β-oxidation pathway. The *Syntrophomonas* BUT1 genome encodes 6 acyl coenzyme A (acyl-CoA) transferases, 7 acyl-CoA dehydrogenases, 8 enoyl-CoA hydratases, 5 3-hydroxybutyryl-CoA dehydrogenases, and 10 acetyl-CoA acetyltransferases (Data Set S2). The presence of homologous β-oxidizing genes was also observed in the type strain Syntrophomonas wolfei subsp. *wolfei* Göttingen DSM 2245B (27). The large number of homologous β-oxidizing genes may afford *Syntrophomonas* BUT1 flexibility to metabolize multiple fatty acid substrates, as its genomic population was detected in heavy [^13^C]DNA during SIP with both [^13^C]butyrate (C_4_) and [^13^C]oleate (C_18_) (11). The different homologous β-oxidizing genes may also have different kinetics and/or affinities, which may allow *Syntrophomonas* BUT1 to adapt to various substrate concentrations. Fluctuating environments are thought to lead to robustness toward gene loss within metabolic networks through an increase in multifunctional enzymes (28). Thus, the presence of various homologous genes for β-oxidation in *Syntrophomonas* BUT1 may have been selected for by fluctuating fatty acid concentrations, such as those imposed from pulse-feeding the anaerobic digester (13). It is also possible that the *Syntrophomonas* BUT1 population was enriched in ^13^C from labeled oleate due to cross-feeding of shorter-chain intermediates during β-oxidation of the C_18_ LCFA, as other syntrophic bacteria were enriched to a high degree during growth on [^13^C]oleate (11). Yet, the enrichment of *Syntrophomonas* BUT1 on [^13^C]butyrate, along with the presence of the complete butyrate β-oxidation pathway, strongly suggests that it is at least capable of β-oxidizing shorter-chain fatty acids (e.g., C_4_) produced in anaerobic environments.

10.1128/mSystems.00159-19.8 DATA SET S2 Annotation information for key metabolic genes for the MAG of *Syntrophomonas* BUT1, including those for beta-oxidation, electron transfer, and others. Download Data Set S2, XLSX file, 0.02 MB. Copyright © 2019 Ziels et al. 2019 Ziels et al. This content is distributed under the terms of the Creative Commons Attribution 4.0 International license.

10.1128/mSystems.00159-19.9 DATA SET S3 Annotation information for key metabolic genes for the MAG of *Methanothrix* BUT2, including those for methanogenesis and others. Download Data Set S3, XLSX file, 0.02 MB. Copyright © 2019 Ziels et al. 2019 Ziels et al. This content is distributed under the terms of the Creative Commons Attribution 4.0 International license.

*Syntrophomonas* BUT1 lacks genes for aerobic or anaerobic respiration, which is similar to genomes of *S. wolfei* and Syntrophus aciditrophicus that are capable of syntrophic butyrate degradation (27, 29). Electrons derived from butyrate oxidation (reduced electron-transferring flavoprotein [ETF] from butyryl-CoA oxidation and NADH from 3-hydroxybutyryl-CoA oxidation) must be disposed of through reduction of CO_2_ to formate and H^+^ to H_2_ via formate dehydrogenases and hydrogenases, respectively (30–33). In the *Syntrophomonas* BUT1 genome, we identified genes encoding butyryl-CoA dehydrogenase, ETF alpha and beta units (EtfAB), and two EtfAB:quinone oxidoreductases (Data Set S2), indicating that this organism may transfer electrons from butyryl-CoA oxidation into membrane electron carriers using ETF. The *Syntrophomonas* BUT1 genome contains five gene clusters encoding formate dehydrogenases and four gene clusters encoding hydrogenases (Data Set S2). These include a membrane-bound cytochrome *b*-dependent selenocysteine-containing formate dehydrogenase and [NiFe] hydrogenase, which may receive butyrate-derived electrons via menaquinol (30). The quinone-binding site of the selenocysteine-containing formate dehydrogenase was on the cytoplasmic side, indicating that it likely utilizes proton motive force to drive unfavorable electron transfer to CO_2_-reducing formate generation outside the cell. Energy investment via “reverse electron transport” is critical to drive the uphill electron transfer from the butyryl-CoA/crotonyl-CoA couple to CO_2_/formate or H^+^/H_2_ couples. In contrast, the quinone binding site of the [NiFe] hydrogenase was on the periplasmic side, indicating that it couples outward vectorial proton transport with H_2_ generation. Previous genomic and proteomic studies also highlight the importance of ETF-based electron transfer, membrane-bound formate dehydrogenases/hydrogenases, and reverse electron transport (5, 27, 33–36).

To complete syntrophic butyrate oxidation, NAD^+^ must also be regenerated through oxidation of NADH. However, NADH oxidation coupled with CO_2_/H^+^-reducing formate/H_2_ generation is thermodynamically unfavorable. To address this obstacle, anaerobic organisms are known to utilize electron bifurcation (or confurcation), which involves the coupling of endergonic and exergonic redox reactions to circumvent energetic barriers (37). For instance, Thermotoga maritima utilizes a trimeric hydrogenase to couple the endergonic production of H_2_ from NADH with the exergonic production of H_2_ from reduced ferredoxin (38). Two trimeric formate dehydrogenase- and two trimeric [FeFe] hydrogenase-encoding gene clusters in *Syntrophomonas* BUT1 appear linked to NADH, as they all contained an NADH:acceptor oxidoreductase subunit (Data Set S2). Yet, if the trimeric hydrogenases and formate dehydrogenases in *Syntrophomonas* BUT1 produce H_2_/formate via electron bifurcation with NADH and ferredoxin, it remains unknown how *Syntrophomonas* BUT1 regenerates reduced ferredoxin, as the known butyrate β-oxidation pathway does not generate reduced ferredoxin (30). Moreover, the *Syntrophomonas* BUT1 genome does not encode an Rnf complex, which would be necessary to generate reduced ferredoxin from NADH. Recently, the Fix (homologous to ETF) system was shown to perform electron bifurcation to oxidize NADH coupled with the reduction of ferredoxin and ubiquinone during N_2_ fixation by Azotobacter vinelandii (39). The *Syntrophomonas* BUT1 genome encoded a Fix-related ETF-dehydrogenase, FixC, as well as its associated ferredoxin, FixX (Data Set S2). A Fix system has also been detected in S. wolfei and was postulated to serve as a means of generating reduced ferredoxin for H_2_ or formate production via the bifurcation mechanism (30). Yet, reduced ferredoxin production with the Fix system would be energetically costly, especially with regard to the low energy yields during syntrophic butyrate oxidation (40). Another mechanism was proposed for generating reduced ferredoxin in Rnf-lacking syntrophs that involves a heterodisulfide reductase complex (HdrABC) and an ion‐translocating flavin oxidoreductase (Flx or Flox) (41). The *flxABCD‐hdrABC* gene cluster was shown to be widespread among anaerobic bacteria, and the protein cluster (FlxABCD-HdrABC) is proposed to function similarly to the heterodisulfide reductase (HdrABC)–[NiFe]-hydrogenase (MvhADG) complex (HdrABC-MvhADG) involved in flavin-based electron bifurcation in hydrogenotrophic methanogenic archaea that couples the exergonic reduction of CoM‐CoB heterodisulfide (CoM‐S‐S‐CoB) with the endergonic reduction of ferredoxin with H_2_ (42). A full *floxABCD‐hdrABC* gene cluster was detected in the genome of *Syntrophomonas* BUT1 (Data Set S2). During the syntrophic growth of *Syntrophomonas* BUT1 on butyrate, the FlxABCD-HdrABC protein cluster may oxidize NADH with reduction of ferredoxin along with the reduction of a high‐redox‐potential disulfide acceptor (42). In Desulfovibrio vulgaris, it has been proposed that the DsrC protein serves as the high‐redox thiol-disulfide electron carrier that is reduced by the FlxABCD-HdrABC complex during growth (43). The DsrC protein was also detected in the syntrophic benzoate-degrading Syntrophorhabdus aromaticivorans strain UI, along with an *flxABCD‐hdrABC* gene cluster (41), suggesting that the reduction of a thiol-disulfide electron carrier may be a conserved mechanism for generating reduced ferredoxin in syntrophic bacteria. Yet, the *Syntrophomonas* BUT1 genome does not encode a DsrC protein, and thus an alternative and unknown thiol-disulfide electron carrier would be needed. Another possibility is that the trimeric hydrogenase can drive NADH-dependent H_2_ generation, as shown in *S. wolfei* Goettingen (40). Nonetheless, this genomic analysis demonstrates that *Syntrophomonas* BUT1 has the potential capacity to overcome energetic barriers during syntrophic butyrate β-oxidation and contains multiple possible mechanisms for H_2_ and formate production.

In addition to interspecies electron transfer via molecular hydrogen and formate, a potential mechanism has been proposed for direct interspecies electron transfer (DIET), in which electrons are shared via electrically conductive nanowires (44). DIET activity has been suggested in enrichment communities degrading propionate and butyrate, in which *Syntrophomonas* was detected (45, 46). However, DIET has not been demonstrated with pure cultures of *Syntrophomonas* to date. The direct transfer of electrons is thought to depend on electrically conductive type IV pili and external polyheme cytochromes (47, 48). The *Syntrophomonas* BUT1 genome encodes a type IV pilin assembly protein, PilC, but no genes that encoded the structural protein PilA, which is associated with DIET (48), were found. Moreover, the type IV pilin genes identified in the *Syntrophomonas* BUT1 genome were of the type Flp (fimbrial low molecular protein weight), which are smaller than the Pil*-*type pilin utilized for DIET in *Geobacter* (49, 50). A multiheme *c*-type cytochrome was detected in the *Syntrophomonas* BUT1 genome that had 59% amino acid identity (89% coverage) with the multiheme *c*-type cytochrome OmcS from G. sulfurreducens, which has been implicated in DIET (48) (Data Set S2). However, that gene also had higher homology (69% identity, 94% coverage) with the cytochrome *c* nitrite reductase from *S. wolfei* (GenBank accession no. WP_081424886). Therefore, the roles of DIET in the metabolism of *Syntrophomonas* BUT1 remain unclear but warrant further attention via expression-based profiling.

In addition to encoding potential genetic mechanisms for energy conservation during syntrophic growth, *Syntrophomonas* BUT1 encodes a capsule biosynthesis protein (CapA), which appears to be specific to syntrophic growth (51). The function of CapA in syntrophic growth is unclear but may be related to the production of exopolymeric substances that facilitate interactions with methanogenic partners (51). The *Syntrophomonas* BUT1 genome also contains the *ftsW* gene, which is related to shape determination and is also a postulated biomarker of a syntrophic lifestyle (51). Based on the presence of these “syntrophic biomarkers” along with genes for β-oxidization and H_2_/formate production, the genomic repertoire of *Syntrophomonas* BUT1 aligns with that of a syntrophic butyrate degrader.

The genome of *Syntrophomonas* BUT1 was compared with published genomes of the *Syntrophomonas* genus (*S*. *wolfei* subsp. *wolfei*, *S. wolfei* subsp. *methylbutyratica*, and *S. zehnderi*) to investigate whether metabolic genes for beta-oxidation and energy conservation were conserved (Data Set S4). Cutoffs of 42% amino acid similarity and 80% sequence overlap were employed based on the lowest first-quartile amino acid similarity that we observed for top BLAST hits (minimum of 20% amino acid similarity and 80% overlap) of *Syntrophomonas* BUT1 genes to each aforementioned *Syntrophomonas* genome (42.0%, 43.5%, and 43.5%, respectively). Based on these similarity thresholds, only 34% (1,050 out of 3,066) of protein-coding genes in the *Syntrophomonas* BUT1 genome have closely related homologs present in all of the other sequenced *Syntrophomonas* genomes. Notably, 40% of the *Syntrophomonas* BUT1 protein-coding genes have no homologs in other *Syntrophomonas* genomes that meet the similarity criteria described above. Reflecting this genomic diversity, *Syntrophomonas* BUT1 encodes several beta oxidation-related genes that have no homologs in the other *Syntrophomonas* genomes that meet the above criteria: one acetyl-CoA acetyltransferase, acyl-CoA dehydrogenase, acrylyl-CoA reductase, and acyl-CoA thioesterase (Data Set S4). In addition, the *Syntrophomonas* BUT1 genome harbors putative isobutyryl-CoA mutase genes (SYNMBUT1_v1_1780025–27) highly similar to those of Syntrophothermus lipocalidus (65.0 to 83.4% amino acid similarity), suggesting that *Syntrophomonas* BUT1 may also be capable of syntrophic isobutyrate degradation. Hydrogenases, formate dehydrogenases, and energy conservation genes were generally conserved among *Syntrophomonas* BUT1 and the other *Syntrophomonas* genomes. Only the cytochrome *b*-dependent [NiFe] hydrogenase has no homologs in the *S. wolfei* subsp. *wolfei* genome. This implies that *Syntrophomonas* BUT1 may have distinct capabilities for fatty acid oxidation, but the levels of energy conservation necessary to drive syntrophic beta oxidation may not vary between *Syntrophomonas* species.

10.1128/mSystems.00159-19.10 DATA SET S4 Genomic comparison of published *Syntrophomonas* BUT1 genomes of the *Syntrophomonas* genus (*S*. *wolfei* subsp. *wolfei*, *S. wolfei* subsp. *methylbutyratica*, and *S*. *zehnderi*). Homologues were identified with a cutoff of 42% amino acid similarity and 80% sequence overlap. Download Data Set S4, XLSX file, 0.2 MB. Copyright © 2019 Ziels et al. 2019 Ziels et al. This content is distributed under the terms of the Creative Commons Attribution 4.0 International license.

A genomic analysis of the *Methanothrix* BUT2 genome indicated that it contained the complete pathway for methane production from acetate (Fig. 4; Data Set S3). This observation agrees with the physiology of other *Methanothrix* species, which are known acetoclastic methanogens (52, 53). *Methanothrix* BUT2 also contained genes that likely are involved in energy conservation during acetoclastic methanogenesis. The genome of *Methanothrix* BUT2 harbored acetyl-CoA synthetase for acetate activation, bifunctional CO dehydrogenase/acetyl-CoA synthase (CODH/ACS) to oxidatively split acetyl-CoA into CO_2_ and CH_3_-H_4_MPT, tetrahydromethanopterin *S*-methyltransferase, and methyl-CoM reductase for methyl-CoM reduction to CH_4_ (Data Set S3). To couple acetyl-CoA oxidation and reductive CH_4_ generation, BUT2 must transfer electrons from reduced ferredoxin to coenzyme M (CoM-SH) and coenzyme B (CoB-SH). We identified an FpoF-lacking F_420_H_2_ dehydrogenase (Fpo) complex and heterodisulfide reductase (HdrDE) that could facilitate this (Data Set S3) and also generate an ion motive force (54). This energy conservation system is highly similar to *Methanothrix thermophila* acetate oxidation (54). In previous studies, *Methanothrix* species have been observed to cooccur with *Syntrophomonas* in LCFA-degrading (13) and butyrate-degrading (55–57) anaerobic environments. In this study, the stable-isotope-informed metagenomic analysis strongly suggests that the labeling of *Methanothrix* BUT2 DNA was due to the incorporation of [^13^C]acetate produced during the degradation of [^13^C]butyrate by *Syntrophomonas* BUT1.

A nearly complete pathway for methane production from CO_2_ was also observed in the *Methanothrix* BUT2 genome (Data Set S3). The only gene lacking in the CO_2_-reducing pathway was an F_420_-dependent *N*^5^,*N*^10^-methylene-tetrahydromethanopterin dehydrogenase (Mtd). While *Methanothrix* is thought to be an obligate acetoclastic methanogen (52, 53), the presence and expression of the CO_2_-reducing pathway in *Methanothrix* were previously reported (58–60) and were hypothesized to be involved in methane formation via DIET. However, the mechanism through which *Methanothrix* directly accepts electrons from its syntrophic partner has not been identified (58, 59). The other known electron donors for methane production from CO_2_ are hydrogen and formate. A membrane-bound hydrogenase (*mbhAB*) was observed in the *Methanothrix* BUT2 genome (Data Set S3). In other studies, negligible hydrogenase activity was observed with *Methanothrix* species (61). Two monomeric formate dehydrogenase enzymes (FdhA) were also encoded by *Methanothrix* BUT2 (Data Set S3). Experiments with thermophilic *Methanothrix* sp. strain CALS-1 and mesophilic Methanothrix concilii showed that they displayed formate dehydrogenase activity by splitting formate into hydrogen and CO_2_; however, the produced CO_2_ was not used for methane generation (61, 62). Yet, the mesophilic *M. soehngenii* did not show formate dehydrogenase activity (53). Thus, the roles of the hydrogenases, formate dehydrogenases, and CO_2_-reducing pathway for methane generation in *Methanothrix* BUT2 are not clear. Transcriptomic, metabolomic, and/or proteomic approaches are needed to elucidate the activity of the CO_2_-reducing methanogenesis production pathway during syntrophic growth on butyrate with *Syntrophomonas* BUT1.

### Conclusions.

In this study, stable-isotope-informed genome-resolved metagenomics was used to provide genomic insights into syntrophic metabolism during butyrate degradation in anaerobic digesters. The results obtained via genome binning and metabolic reconstruction showed that a ^13^C-enriched *Syntrophomonas* genome contained the genetic capacity to convert butyrate into precursor metabolites for methane formation: acetate, hydrogen, and formate. A ^13^C-enriched *Methanothrix* genome likely consumed the acetate produced during butyrate degradation, incorporating some ^13^C into biomass. The presence of a CO_2_-reducing pathway, as well as formate dehydrogenase and hydrogenase genes, in the *Methanothrix* genome leaves open the possibility of flexible metabolism during methanogenesis. As syntrophic fatty acid-degrading populations are often slow-growing and thus difficult to isolate, this study demonstrates a new approach to link ecophysiology with genomic identity in these important populations involved in anaerobic biotechnologies as well as global carbon cycling. Advancing our understanding of *in situ* metabolic activities within anaerobic communities is paramount, as these microbiomes contain multiple interacting functional groups that, in cooperation, enable the processing of degradable organic carbon into methane gas. Coupling SIP-informed metagenomics with other activity-based techniques, such as metabolomics, transcriptomics, and proteomics, may further illuminate the structure of anaerobic metabolic networks as well as quantify metabolite fluxes, thus enabling newly informed process models to predict rates of anaerobic carbon transformation.

## MATERIALS AND METHODS

### Batch incubations with [^13^C]butyrate.

Two 4-liter anaerobic digesters treating dairy manure and sodium oleate were operated for over 200 days at a solids retention time of 20 days and a temperature of 35°C, as described by Ziels et al. (13). The two digesters were operated with different feeding frequencies of sodium oleate. One digester received sodium oleate once every 48 h, while the other digester was fed semicontinuously every 6 h (13).

On day 228 of digester operation, 10-ml samples were collected from each digester and immediately transferred to 35-ml glass serum bottles that were prepurged with N_2_-CO_2_ (80:20) and capped with butyl rubber septa. At the time of biomass sampling, total effluent volatile fatty acids (VFA) and LCFA (liquid plus sorbed) levels were below 70 mg/liter. Duplicate microcosms were fed with a 1 M solution of either ^12^C sodium butyrate or ^13^C-labeled sodium butyrate (>98% atom purity; Cambridge Isotope Laboratories, Tewksbury, MA, USA) to reach an initial butyrate concentration of 40 mM. The ^13^C-labeled sodium butyrate was universally labeled at all 4 carbons. Triplicate blank controls were incubated in parallel to measure background methane production from the inoculum. Methane production was measured approximately every 4 h over the 50-h incubation time using a digital manometer (series 490 A; Dwyer Instruments) and gas chromatograph-flame ionization detector (GC-FID) (item no. SRI 8610C), according to the methods of Ziels et al. (13). Cumulative methane production from butyrate degradation was determined by subtracting the cumulative methane production in unamended controls over time. A 50-h incubation time was used to limit cross-labeling of peripheral populations with by-products of endogenous decay (11, 12), while also providing sufficient time for nearly all of the substrate (>80%) to be converted.

### Stable-isotope probing.

DNA was extracted from the duplicate 10-ml microcosms after the 50-h incubation, separated via density gradient centrifugation, fractionated, precipitated, and recovered as previously described (11). DNA was measured in 24 density gradient fractions using Qubit (Invitrogen, MA, USA). *Syntrophomonas* 16S rRNA genes were quantified in gradient fractions as described by Ziels et al. (11), using previously developed primers and probes (63). Heavy-DNA fractions with buoyant densities between 1.70 and 1.705 g/ml (see Fig. S2 in the supplemental material) were selected for each microcosm sample and sent for metagenomic sequencing at MR DNA Laboratories (Shallowater, TX, USA), as well as for 16S rRNA gene iTag sequencing at the U.S. Department of Energy Joint Genome Institute (JGI), according to the method of Ziels et al. (11). Metagenome libraries were prepared using the Nextera DNA sample preparation kit (Illumina Inc., Hayward, CA, USA) by following the manufacturer’s instructions. The metagenome libraries were sequenced in 150-bp paired-end mode on a HiSeq 2500 sequencer (Illumina Inc., Hayward, CA, USA).

### 16S rRNA gene amplicon sequence analysis.

Raw 16S rRNA gene amplicon reads were filtered by trimming the first 10 bp, truncating forward reads at 265 bp, truncating reverse reads at 180 bp, and filtering all reads based on a maximum expected error of 2 using DADA2 (64). The filtered and trimmed reads were then dereplicated and denoised into exact sequences using estimated error parameters with DADA2. Forward and reverse sequences were then merged with DADA2 using a minimum overlap of 20 bp and zero allowed mismatches. Merged and denoised sequences were then truncated to 390 bp and clustered into OTUs with a 99.5% similarity cutoff after chimera removal with UPARSE v.8.1 (65). Representative sequences of the 99.5% OTUs were classified against those in the SILVA SSU Ref nonredundant data set, v.123, using the RDP classifier (66).

### Metagenome binning, annotation, and statistical analysis.

All metagenomic reads were initially trimmed and quality filtered using illumina-utils (67) (available from https://github.com/merenlab/illumina-utils ) according to the parameters of Minoche et al. (68). Metagenomic reads from all [^13^C]butyrate-fed microcosms were coassembled using MEGAHIT v1.1.1 (69). Open reading frames were called with Prodigal v.2.6.3 (70) and were taxonomically classified with GhostKOALA (71). Short reads from the ^12^C and ^13^C metagenomes were mapped onto the contigs using Bowtie 2 (72) with default parameters and parsed with SAMtools v.1.3.1 (73). Additionally, bulk community metagenomic reads from the total biomass collected from each digester within 2 days of the butyrate SIP incubations were mapped onto the assembled contigs for their inclusion in the subsequent differential-coverage binning. The contigs were then binned according to the workflows of Eren et al. (74) and Lee et al. (75) using anvi’o v.2.4.0 and CONCOCT v.1.0.0 (76). Briefly, single-copy genes were searched using the “anvi-run-hmms” command. Single-copy genes were identified using hidden Markov models in anvi’o based on the Campbell et al. (77) and Rinke et al. (78) bacterial and archaeal gene data sets, respectively. The “anvi-profile” command was used to parse contig coverage across all samples from the BAM files with SAMtools (73). The “anvi-merge” command was used to compile the coverage information for contigs across all samples into a single anvi’o profile. The initial binning was conducted with the “anvi-cluster-with-concoct,” which uses CONCOCT (76), by constraining the number of bins to 40 (“–num-clusters 40”) to minimize fragmentation error (i.e., splitting up a single bin into multiple smaller bins) (75). Bins that displayed “conflation error” (i.e., a bin has multiple populations and/or contamination) (75) were interactively refined using the “anvi-refine” command based on completion and redundancy estimates from the presence of bacterial and archaeal single-copy genes, taxonomies of open reading frames (ORFs) from BlastKOALA, tetra-nucleotide frequency, and coverage patterns across multiple samples. After manual refinement of the bins using anvi’o, we obtained a set of 160 genomic bins that were assessed for completeness and contamination with CheckM (20) (Data Set S1). The differential abundance of each genomic bin in the [^13^C]- and [^12^C]butyrate metagenomes of each digester was determined using DESeq2 (14) using mapped read counts. A significant difference in abundance between ^12^C and ^13^C metagenomes was established by a *P* value of less than 0.05. The average nucleotide identity (ANI) between ^13^C-enriched genomic bins and publicly available genomes from closely related organisms was calculated with pyANI (available from https://github.com/widdowquinn/pyani ). Open reading frames were annotated with the MicroScope platform (79), and metabolic reconstructions were performed with Pathway Tools (80). Potential type IV pilin genes were identified with the PilFind program (49).

### Data availability.

We have made publicly available the following: raw sequence reads and metagenome assemblies for the butyrate DNA-SIP metagenomes in NCBI’s Sequence Read Archive under BioProject no. PRJNA524401, genomic FASTA files for each ^13^C-enriched genomic bin (https://doi.org/10.6084/m9.figshare.7761776), and the annotation data for the two ^13^C-enriched MAGs (https://doi.org/10.6084/m9.figshare.7761710). The bulk community raw metagenomic reads from the study by Ziels et al. (11) that were used in differential coverage binning are available via the U.S. Joint Genome Institute’s Genome Portal (https://genome.jgi.doe.gov/portal/) under the project identifiers 1105507 and 1105497. 16S rRNA gene amplicon sequences are available via the U.S. Joint Genome Institute’s Genome Portal under project no. 1105527, with sample identifiers 112232 to 112239.
