# Supplementary figures and images for: Elucidating Syntrophic Butyrate-Degrading Populations in Anaerobic Digesters Using Stable-Isotope-Informed Genome-Resolved Metagenomics
Source: mSystems. 2019 Aug 6;4(4):e00159-19. doi: 10.1128/mSystems.00159-19 (PMC6687939; doi:10.1128/mSystems.00159-19)

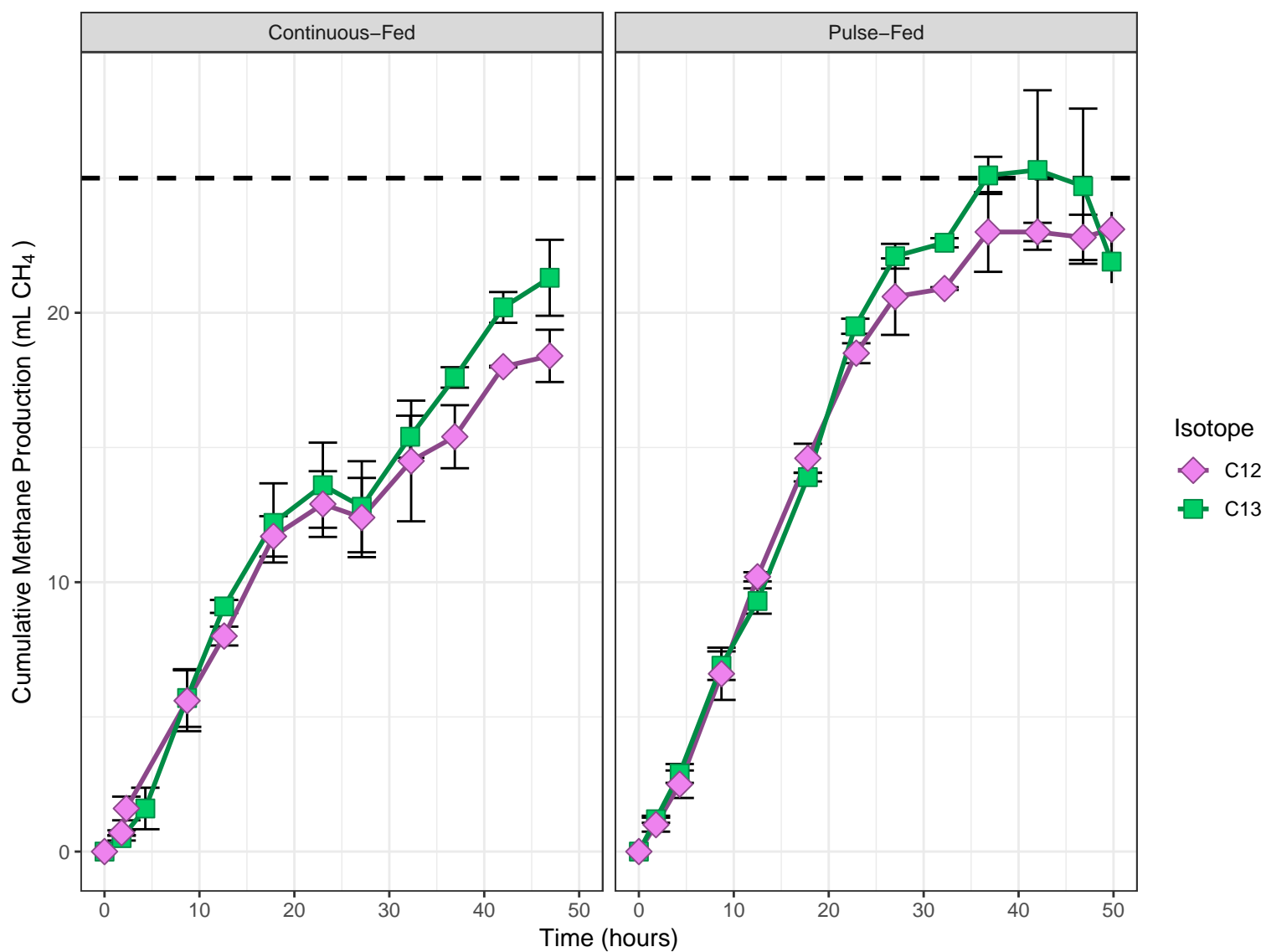

Supplement: FIG S1 [file mSystems.00159-19-sf001.pdf]

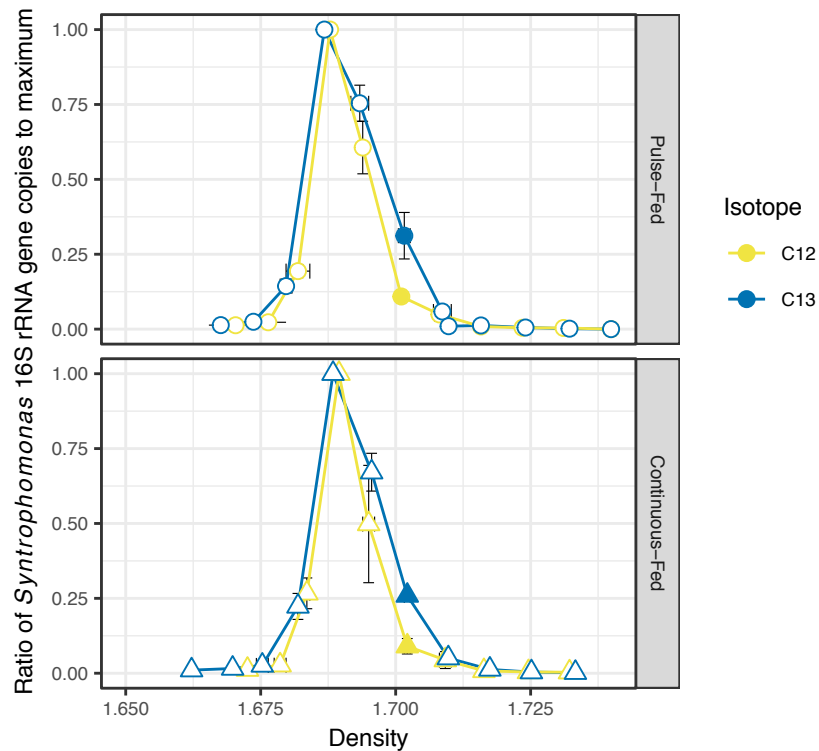

Supplement: FIG S2 [file mSystems.00159-19-sf002.pdf]

A

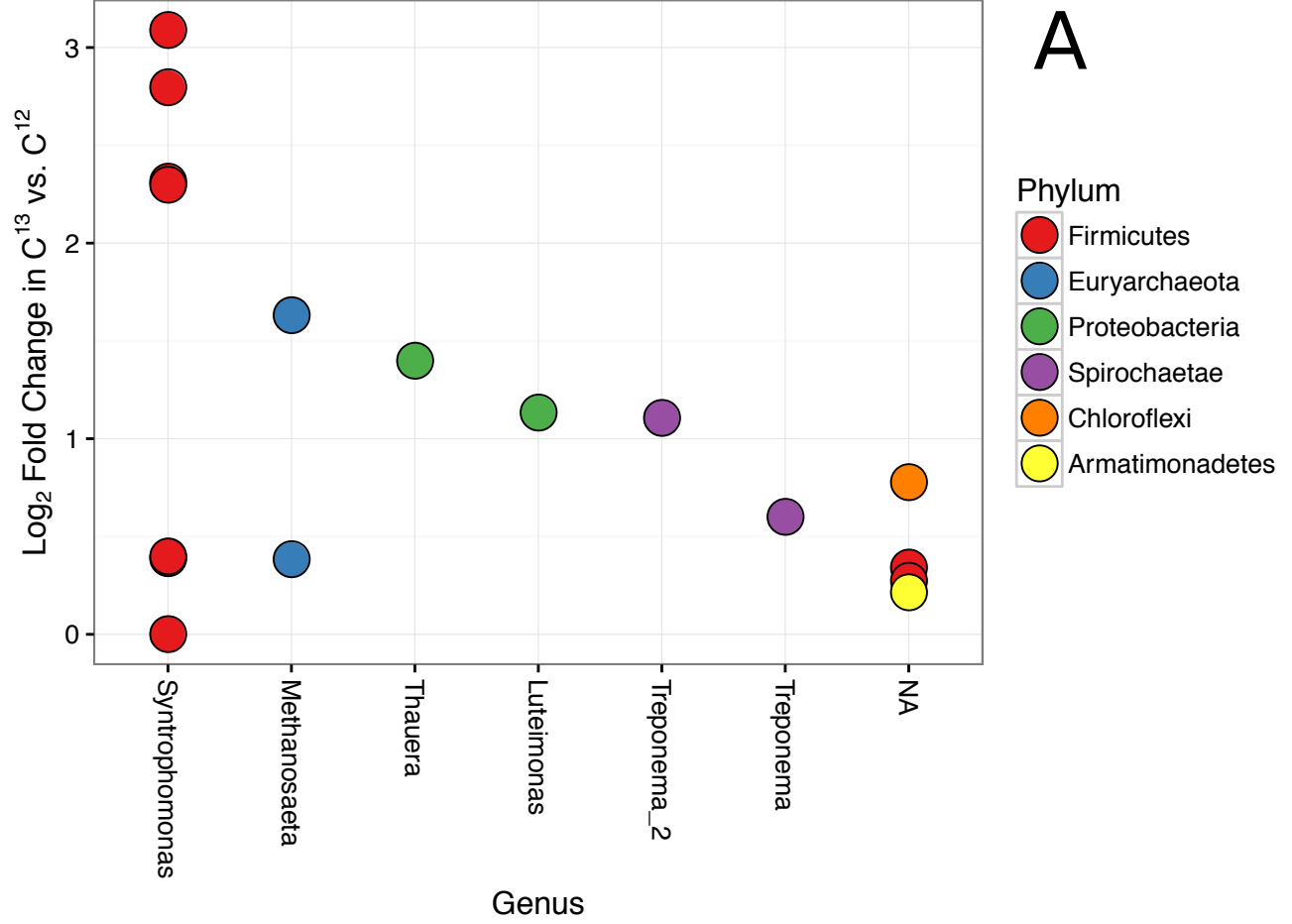

B

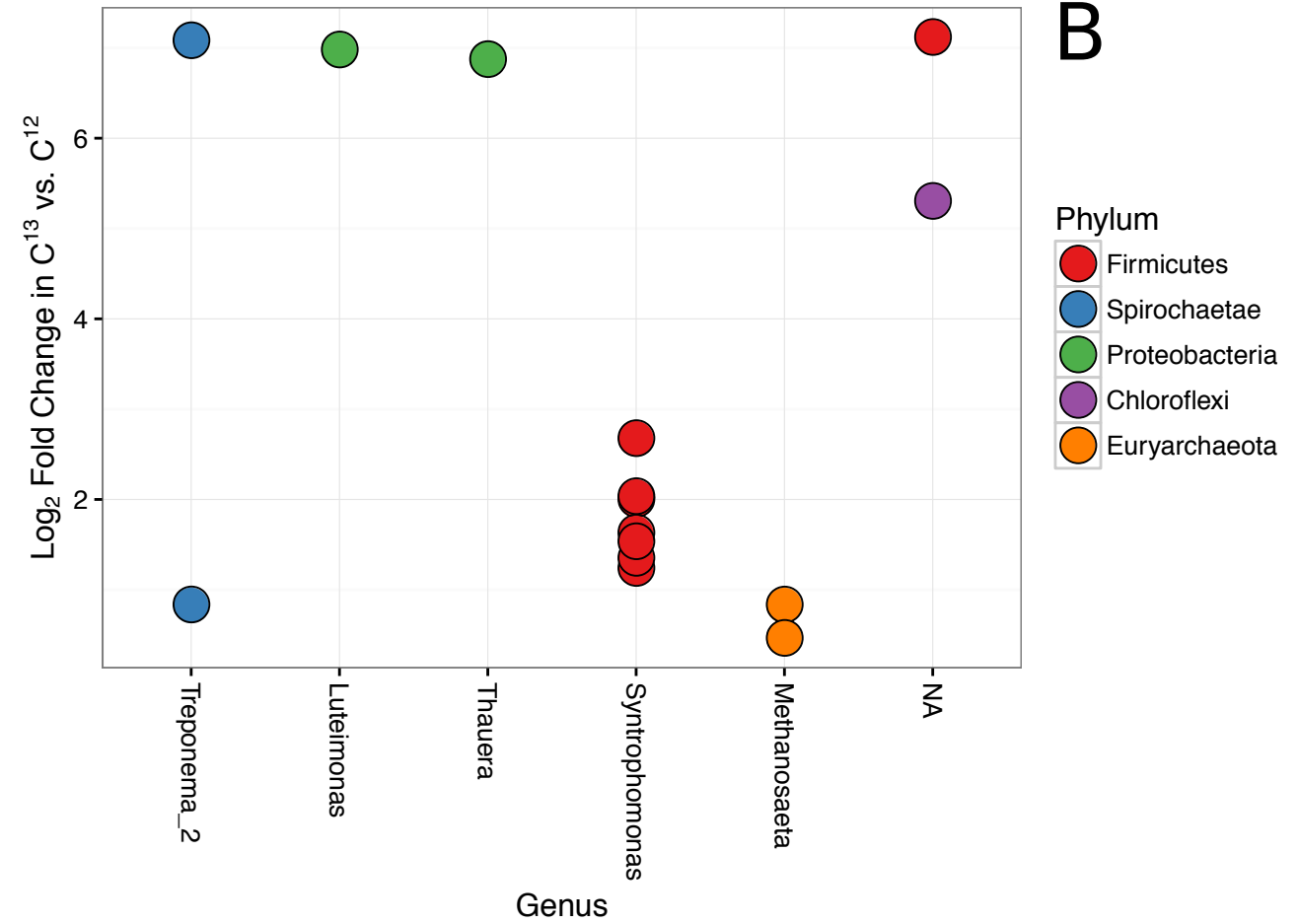

Supplement: FIG S3 [file mSystems.00159-19-sf003.pdf]

A

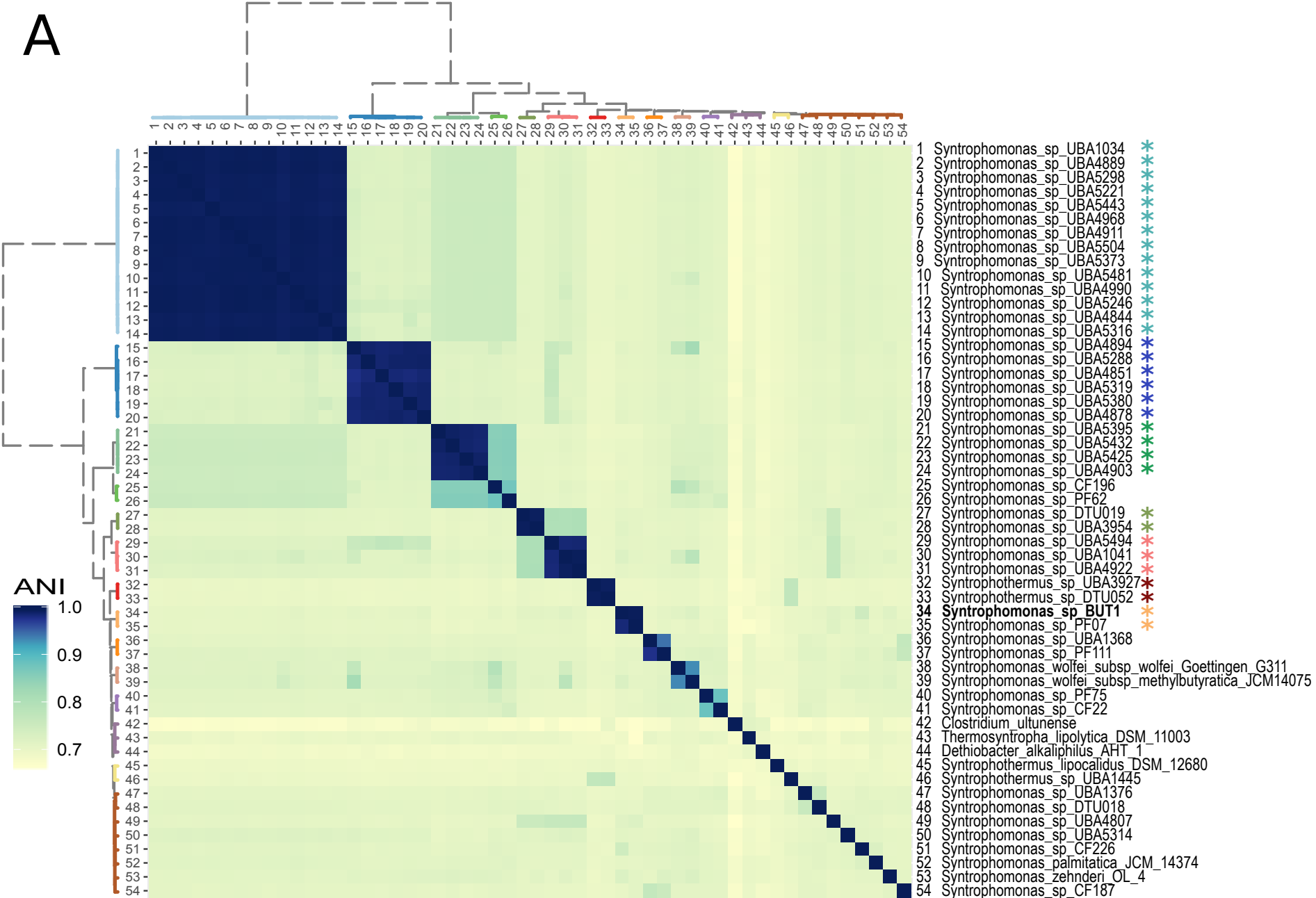

B

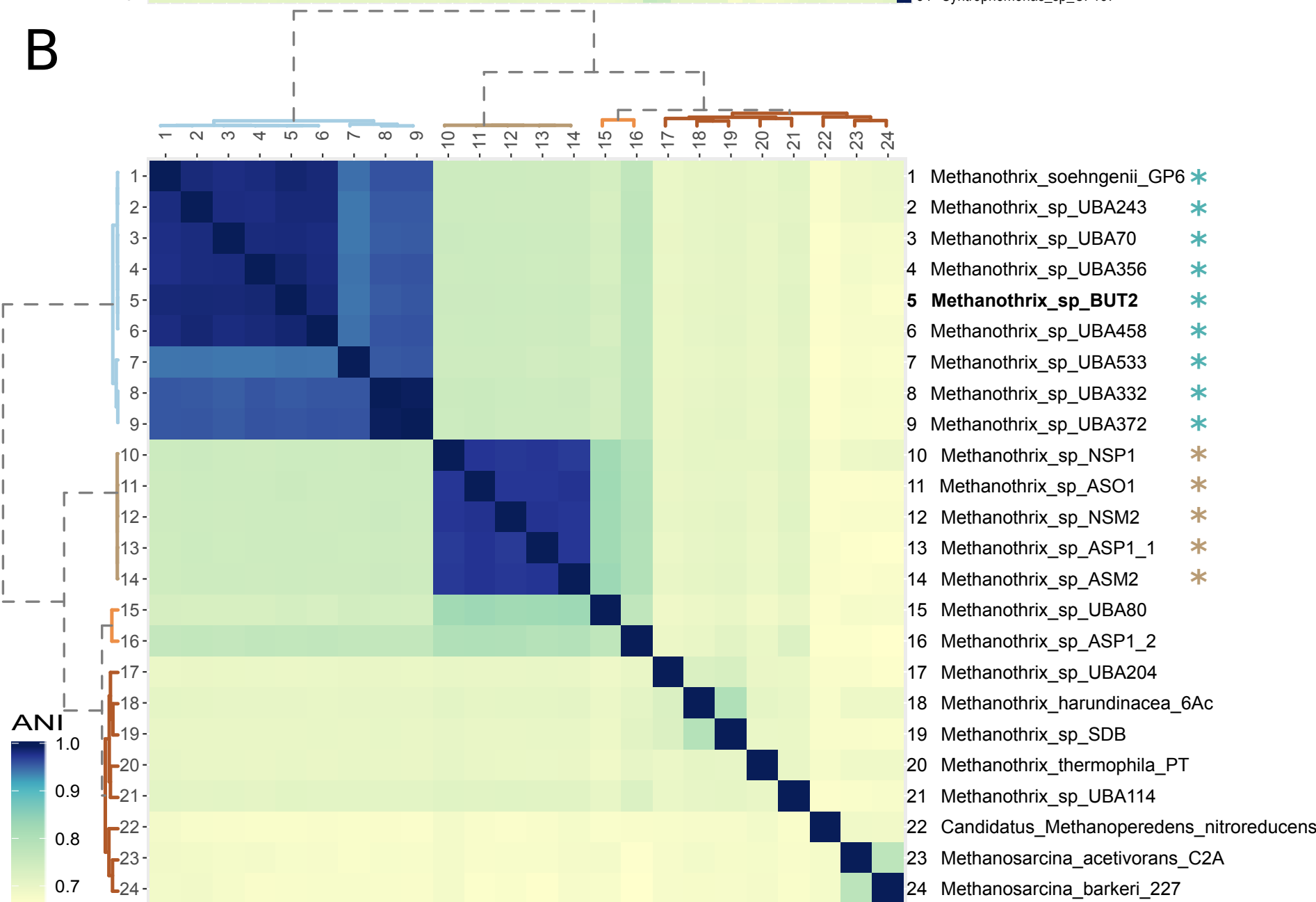

Supplement: FIG S4 [file mSystems.00159-19-sf004.pdf]
