# Supplementary material for: Elucidating Syntrophic Butyrate-Degrading Populations in Anaerobic Digesters Using Stable-Isotope-Informed Genome-Resolved Metagenomics
Source: mSystems. 2019 Aug 6;4(4):e00159-19. doi: 10.1128/mSystems.00159-19 (PMC6687939; doi:10.1128/mSystems.00159-19)
Supplement: TABLE S1 [file mSystems.00159-19-st001.docx]

| **Digester** | **Replicate** | **Isotope** | **Raw Reads** | **Filtered Reads** | **Reads Mapped to Co-assembly (%)** |
| --- | --- | --- | --- | --- | --- |
| Pulse Fed | A | ^12^C | 31,425,432 | 28,097,374 | 63.98 |
| Pulse Fed | B | ^12^C | 31,646,858 | 28,395,122 | 64.14 |
| Pulse Fed | A | ^13^C | 40,852,542 | 36,232,668 | 68.16 |
| Pulse Fed | B | ^13^C | 25,672,716 | 23,368,004 | 69.12 |
| Continuous Fed | A | ^12^C | 36,841,972 | 32,975,712 | 69.97 |
| Continuous Fed | B | ^12^C | 35,962,594 | 32,641,070 | 66.65 |
| Continuous Fed | A | ^13^C | 29,134,756 | 26,823,396 | 69.34 |
| Continuous Fed | B | ^13^C | 34,718,070 | 31,132,802 | 70.26 |
