# Supplementary material for: Elucidating Syntrophic Butyrate-Degrading Populations in Anaerobic Digesters Using Stable-Isotope-Informed Genome-Resolved Metagenomics
Source: mSystems. 2019 Aug 6;4(4):e00159-19. doi: 10.1128/mSystems.00159-19 (PMC6687939; doi:10.1128/mSystems.00159-19)
Supplement: TABLE S2 [file mSystems.00159-19-st002.docx]

| **Reaction** | | ***Δ_r_G'°*** | ***Δ_r_G'* ^1^** |
| --- | --- | --- | --- |
| *Acetogenic Reactions* | | | |
| (1) | Butyrate^-^ + 2 H_2_O ⇌ 2 Acetate^-^ + 2 H_2(g)_ + H^+^ | +53.1 ± 13.6 | -21.1 ± 13.6 |
| (2) | Oleate^−^ + 16 H_2_O ⇌ 9 acetate^−^ + 15 H_2(g)_ + 8H^+^ | +344.9 ± 91.5 | -219.9 ± 91.5 |
| *Methanogenic Reactions (Butyrate oxidation)* ^2^ | | | |
| (3) | 2 H_2(g)_ + 1/2 CO_2(g)_ ⇌ 1/2 CH_4(g)_ + H_2_O | -66.5 ± 12.4 | -9.4 ± 12.4 |
| (4) | 2 Acetate^-^ + 2 H^+^ ⇌ 2 CO_2(g)_ + 2 CH_4(g)_ | -70.3 ± 16.9 | -58.9 ± 16.9 |
| *Methanogenic Reactions (Oleate oxidation)* ^2^ | | | |
| (5) | 15 H_2(g)_ + 15/4 CO_2(g)_ ⇌ 15/4 CH_4(g)_ + 15/2 H_2_O | -498.5 ± 93.0 | -70.6 ± 93.0 |
| (6) | 9 Acetate^-^ + 9 H^+^ ⇌ 9 CO_2(g)_ + 9 CH_4(g)_ | -316.3 ± 75.9 | -264.9 ± 75.9 |
| *Overall Reactions* | | | |
| (7) | Butyrate^-^ + H_2_O + H^+^ ⇌ 3/2 CO_2(g)_ + 5/2 CH_4(g)_ | -83.7 ± 18.0 | -89.4 ± 18.0 |
| (8) | Oleate^−^ + 17/2 H_2_O + H^+^ ⇌ 51/4 CH_4(g)_ + 21/4 CO_2(g)_ | -469.8 ± 81.9 | -555.4 ± 81.9 |

^1^All values are in units of kJ/mole-reaction, and were calculated using eQuilibrator (<http://equilibrator.weizmann.ac.il/>) at 25°C, pH of 7 and ionic strength of 1. *Δ_r_G'* values were calculated based on fatty acid concentrations of 1 mM, H_2_ partial pressures of 1 Pa, and CO_2_ and CH_4_ partial pressures of 10^4^ Pa.

^2^ The stoichiometry of the methanogenic reactions were scaled proportionally to consume the products from oxidizing 1 mole of fatty acid.
